# Revising Parental Burnout Theory: Toward a Differentiation of Sleep-Related Burnout Subtypes

**DOI:** 10.3390/children13030394

**Published:** 2026-03-12

**Authors:** Royce Anders, Agnès Breton, Florian Lecuelle, Mélanie Havy, Lisa Brunel, Marie-Paule Gustin, Patricia Franco, Benjamin Putois

**Affiliations:** 1 EPSYLON Laboratory, Department of Psychology, University of Montpellier Paul Valéry, 34000 Montpellier, France; 2 Faculty of Psychology, Unidistance Suisse, 3900 Brig, Switzerland; 3 Lyon Neuroscience Research Centre, CNRS UMR 5292-INSERM U1028, Lyon 1 University, 69100 Villeurbanne, France; 4 Department of Pediatric Clinical Epileptology, Sleep Disorders and Functional Neurology, University Hospitals of Lyon, 69000 Lyon, France; 5 Public Health, Epidemiology and Evolutionary Ecology of Infectious Diseases (PHE3ID), International Centre for Infectiology Research (CIRI), INSERM U1111, CNRS UMR5308, ENS Lyon, 69000 Lyon, France; 6 Public Health Department, Biostatistics, Institute of Pharmaceutic and Biological Sciences, University Claude Bernard Lyon 1, 69100 Villeurbanne, France

**Keywords:** parental burnout, child sleep disturbances, psychosocial risk factors, insomnia, early childhood

## Abstract

Background: Contemporary models of parental burnout conceptualize it as an interplay between parental demands and insufficient resources. However, research and current models remain sparse in their understanding of these demands and dynamics within the context of managing a child’s sleep wellness and related problems, which constitute a fundamental aspect in early parenting. The present work addresses this gap by examining this issue comprehensively. Methods: 2291 mother–child dyads were recruited from two sources: a random population sample (*n* = 1409) and a clinical sample (*n* = 882) of mothers seeking consultation for their child’s sleep issues (0–5 years old). Mothers completed an extensive panel of validated instruments and survey questions covering burnout and psychopathologies, sleep parameters, psychosocial, organizational, and demographic variables. Inferential analyses, regression modeling, cluster analysis, and mediation models were applied. Results: Two distinct profiles of parental burnout emerged: one associated with child sleep disturbances and the other with general parenting stress. The strongest-weighted risk factors pertained to maladaptive beliefs and perceptions (e.g., shame, “I am a bad parent”, “My child cries because I do not meet his needs”), as well as additive stressors such as interparental tension and daytime child behavioral problems. The strongest protective factors involved resources that reduced parental demands or facilitated recovery, including couple satisfaction, a consistent bedtime routine, greater capacity to take breaks (e.g., additional caregivers, father nighttime involvement, parental cohabitation, and child screen time). Conclusion: The identification of two distinct burnout profiles highlights the importance of incorporating, or placing more centrally, the management of young children’s insomnia in contemporary theoretical models of parental burnout. This research highlights the need for interventions on healthy self-beliefs and perceptions, effective daytime parenting strategies, positive couple dynamics, consistency in bedtime routines, and equitable distribution of caregiving responsibilities between parents to reduce the risk of parental burnout.

## 1. Introduction

Parental burnout syndrome arises from a chronic imbalance between the demands faced by parents and the resources available to cope with them [1,2]. It is characterized by intense emotional exhaustion, emotional distancing from one’s children, and a reduced sense of parental accomplishment. This syndrome is distinguished by specific clinical manifestations [3,4,5]. Its prevalence ranges from 5% to 8% in Western populations [6], with a higher incidence among mothers than among fathers [7,8]. Parental burnout carries serious consequences, including an increased risk of child neglect and maltreatment [9], as well as negative impacts on parents’ mental health, such as elevated suicidal ideation and addictive behaviors [10]. The Balance Between Risks and Resources (BR^2^) model is the primary theoretical framework in this field [1]. Developed using a sample of 923 parents, it provides a reliable assessment of the balance between factors that increase parental stress and the resources that mitigate it.

The etiology of parental burnout is multifactorial [1]. Two meta-analyses—one including 26 studies [11] and the other involving 35,170 parents [12]—have shown that parental burnout is associated with a combination of organizational, relational, and individual-level factors. At the organizational level, relevant factors include the number of children, single parenthood, time spent with children, low social support, and difficulties in balancing work and family life, which have been identified as influential factors. At the relational level, challenging parent–child interactions, couple dissatisfaction, and disagreements in coparenting exert an influence. At the individual level, influential factors include personality traits (notably neuroticism, perfectionism, and a need for control), low self-esteem, reduced resilience, poor emotion regulation, anxiety, depression, occupational burnout, and sleep disorders are influential parental factors. Regarding the child, prior research has largely focused on behavioral or health problems that have been identified. Notably, child sleep is a variable that, to our knowledge, has never been taken into account in empirical studies specifically addressing parental burnout. Although an observational study involving of over 14,000 parents [13] found that parental stress is 90% higher when a child has sleep problems, and up to 189% higher in cases of insomnia, these findings refer to parental stress more generally rather than parental burnout per se.

Sleep disorders affect up to 40% of children under five years of age [14,15,16]. In 80% of cases, these are behavioral insomnias that require frequent parental interventions at bedtime and during nighttime awakenings [17]. From the perspective of the BR^2^ model, such disorders constitute a major factor increasing parental demands, not only in terms of intensity but also in terms of duration, by extending parental responsibility demands across a 24 h period.

The impact of child sleep disorders on parental sleep is now well documented [18,19]. Parents of so-called “poor sleepers” may take up to six years to regain a pre-child sleep quality with mothers being particularly affected by this chronic disruption [20]. In line with the BR^2^ model, maternal sleep may therefore be conceptualized as a resource that is compromised by the demands imposed by a child presenting with a sleep disorder. Indeed, parental insomnia is strongly correlated with levels of daytime stress [21]. Furthermore, there also appears to be a bidirectional relationship between parental sleep deprivation and dysregulation of the hypothalamic–pituitary–adrenal axis [3,22].

Large-scale studies indicate that mothers, in as high as 91% of cases, are the primary caregivers responsible for their children’s (i.e., 0–5 years old) sleep wellness in up to 91% of cases [23]. And, consistent with this finding, studies have shown that mothers exhibit a higher average of burnout symptoms than fathers on average [7,8]. Given the higher parental demands faced by many mothers, social support through extended family or shared parenting responsibilities constitutes a critical resource. For example, fathers’ involvement in nighttime caregiving has been shown to be a protective factor against sleep disturbances in both the children [24,25] and mothers [26]. The Cognitive and Emotional Model of Young Children’s Insomnia (CEMYCI) [27] describes how insomnia in young children arises from daytime and nighttime educational challenges and how its etiology is rooted in parents’ dysfunctional emotions and cognitions about their child’s sleep and nighttime crying. Specifically, beliefs that leaving a child alone at night constitutes parental neglect (e.g., being a bad parent) are associated with shame and guilt. These emotions lead parents to intervene at night more than necessary, preventing their children from learning to fall asleep independently. Such maladaptive cognitions, emotions, and behaviors contribute to insomnia in both children and parents and are associated with parental burnout [27]. In line with the BR^2^ model, these factors combined with the previously identified organizational and individual variables provide a solid framework for understanding burnout through the lens of managing child sleep wellness and related problems.

Therefore, the primary objective of this study was to identify the variables most strongly associated with parental (maternal) burnout with a particular focus on child’s (0 to 5 years) sleep wellness and its management. A secondary aim was to account for other maternal psychopathological symptoms. Specifically, burnout symptoms, as well as related pathologies (e.g., insomnia, anxiety, depression) were assessed in a large sample of mother–child dyads, alongside a comprehensive set of organizational, relational, and individual-level variables. These included bedtime routines and nighttime interventions, parenting and child behavioral variables, couple dynamics, parental psychology, perceptions, prior experiences, and demographic variables. This approach allowed for the assessment of a maximal number of variables potentially influencing burnout, while respecting constraints imposed by the sample-size-to-number-of-variables ratio. This study included 2291 mother–child dyads.

Our theoretical hypothesis posits that the sleep patterns of both children and parents exert a strong influence on parental burnout syndrome. In accordance with CEMYCI [27], the primary hypotheses are as follows: (1) mothers of children who sleep poorly will, on average, exhibit higher parental burnout scores; (2) mothers with elevated levels of shame and guilt related to their child’s sleep will have higher parental burnout scores; (3) consistent with the previous hypothesis, mothers who interpret their child’s nocturnal cries as expressions of attachment or pain (and hence unmet needs/demands) will exhibit higher parental burnout scores; (4) mothers with lower levels of social support, whether from their family or their partner, will exhibit higher burnout scores. Two mediation hypotheses are also proposed: first, related to (1), child insomnia may predict maternal insomnia, which in turn predicts parental burnout; and second, related to (2) and (3), higher attachment/pain-related interpretations of crying may predict elevated shame, which subsequently predicts higher burnout. Finally, this study incorporates exploratory, data-driven analyses through modeling (e.g., feature selection and clustering, also referred to as unsupervised learning) to identify instrumentally additional variables that may play a critical role in parental burnout, thereby advancing current understandings in novel ways.

## 2. Materials and Methods

### 2.1. Participants

This study involved a clinical group and a control group. Data was collected between 2019 and 2023. The clinical group consisted of families seeking consultation for childhood sleep disorders at the Dormium Sleep Institute (sommeilenfant.org). All voluntary families between these dates that satisfied the inclusion criteria during this period were accepted for the study, which consisted of 882 participants, and required having a child aged from 0 to 5 years old. The control group was recruited online from the general population through the survey institute Panelabs which ensures representative sampling, yielding 1409 participants, with children within the same age range. In accordance with a prior large-scale study indicating that mothers are predominantly responsive (more frequently) for their child(ren)’s sleep care—that is, at least up to 91% of cases [23]—only mothers were asked to complete the questionnaires.

Only participants who completed all the questionnaires were retained for the analyses, resulting in 100% data completeness in the analyzed sample. This does not represent the response rate among all invited participants. Control group participants received €7 for participation. In the clinical group, families benefited from personalized advice, diagnostic screening, and referrals to qualified professionals specialized in sleep disorders in young children.

### 2.2. Procedure

This study received ethical approval from the Institutional Review Board (Comité de Protection des Personnes, CPP-EST II) with identification number 21.01.18.75329. Participation was voluntary, respondents could withdraw from the study at any time. All data were anonymized prior to analysis and handled in a strictly confidential manner, in accordance with national research regulations specified by the French Data Protection Authority (Commission nationale de l’informatique et des libertés, CNIL) and the ethical principles of the Declaration of Helsinki [28] and its amendments.

### 2.3. Measures

The primary objective of this study was to examine parental burnout scores (maternal), measured using the Parental Burnout Inventory (PBI) [2], and to identify the variables most strongly associated with burnout, across a wide range of parent- and child-related factors assessed. The PBI consists of 22 items rated on a 7-point Likert scale (0–6) assessing emotional exhaustion, emotional distancing, and feelings of personal accomplishment (Cronbach’s α = 0.92). The maximum possible score is therefore 132. A multimethod approach suggests cut-off scores of ≥54 for moderate burnout severity and ≥75 for clinical or impairing burnout levels [29].

Maternal psychological and psychopathological variables were assessed with validated instruments. These included: the Beck Depression Inventory (BDI–13) [30], which measures depression across 13 items rated on four levels (Cronbach’s α = 0.84); the State–Trait Anxiety Inventory (STAI Y-A and Y-B) [31], which assesses state anxiety and trait anxiety across two 20-item scales, respectively (α = 0.89); the Insomnia Severity Index (ISI) [32], consisting of seven items (total score range 0–28) (α = 0.82); the Pichot Fatigue Scale [33], composed of eight items (score range 0–32) (α = 0.95); the Personal Feelings Questionnaire (PFQ–2 Brief) [34], which measures guilt and shame related to children sleep across six and 10 items, each rated on a five-point Likert scale with Cronbach α values of 0.82 and 0.87; the five-item short version of Horn and Östberg’s Morningness–Eveningness Questionnaire (MEQ) [35], evaluating whether the parent’s chronotype is more morning or evening oriented, across items are rated on a five-point Likert scale (α = 0.60); the Baby Crying at Night (BBCN) scale [36], a 14-item questionnaire for which items are rated on a seven-point Likert scale measuring parental beliefs about their young child’s nocturnal crying, including attachment needs, pain, the potential traumatizing effect of crying alone, or the expression of a general need to cry (α = 0.78). Note that mother Anxiety and Depression variables were included to characterize associated symptom profiles and to ensure that the observed burnout patterns were not reducible to general psychological distress. They were not used to define parental burnout nor included as primary predictors in the main regression model.

Children’s sleep disorders were assessed using the Sleep Disturbance Scale for Children (SDSC) [37,38], which evaluates sleep disturbances in children aged 6 months to 4 years through 22 items rated on a five-point scale ranging from “Never” to “Always”. A number of studies have evaluated and/or validated the SDSC on different age ranges such as preschool and prior (up to 6 years old) [37,38] and adolescence (e.g., 6–16 years old) [39,40]. The scale is divided into five dimensions: hyperhidrosis, breathing disorders, parasomnias, non-restorative sleep, excessive daytime sleepiness, and difficulty in initiating and maintaining sleep. The observed Cronbach’s α for the total SDSC score was 0.84. Child behavioral problems were assessed with the Conners’ Parent Rating Scale (CPRS)–abbreviated version which consists of 10 items [41], also referred to in the literature as the 10-item Conners’ Rating Scale. A Cronbach α of 0.88 was observed.

Sociodemographic variables including maternal age, marital status, employment status, and educational level were assessed along with child-related variables such as age category, sex, birth order, feeding method, number of nocturnal awakenings. Daily life variables were also measured including daytime parental involvement, household tasks, parent relationship satisfaction, disagreements regarding parenting values, couple conflict, which parents intervene more often at night for their child, ability to delegate, and level of perfectionism. These variables were assessed using non-standardized items (See Supplementary Data S1 for more details on these items).

### 2.4. Data Transparency and Openness Statement

The dataset contains sensitive patient information and is not posted in a public repository due to legal and ethical constraints. However, it may be requested for research purposes via a data sharing agreement by contacting the corresponding author. This study models the data from [27], focusing specifically on parental burnout, whereas the prior study modeled child insomnia. Additionally, the present study includes a larger set of variables not previously analyzed. Data were performed using Python, version 3.12 [42] and the packages *scipy*, *statsmodels*, and *pingouin*: versions 1.0 [43], 0.14 [44], and 0.5 [45] respectively, as well as the *lavaan* package version 0.6 [46] in R version 4.3.3 [47] for structural equation (or mediation) modeling.

### 2.5. Statistical Analyses

First, to provide an overview of the full sample (2291 participants in total), descriptive statistics were calculated for all relevant variables and questionnaires. Also, internal consistency reliability of the instruments used was assessed using Cronbach’s α, as reported above in Section 2.3. Then, to satisfy the assumptions required for parametric tests, the data were normalized using the non-paranormal transformation [48,49] and subsequently standardized.

Predictors and variables most strongly associated with parental burnout were assessed using linear modeling. Following the univariate inferential tests conducted for the primary hypotheses presented in the Introduction, a data-driven, multivariate approach was applied to identify the set of variables (feature selection) that maximally improved model fit in predicting the PBI, based on the Akaike Information Criterion (AIC) statistic [50,51], using top-down regression. The stability of this feature selection method, and resulting variable weights, were verified across different parameterizations of the fitting method. Similar results compared to alternative approaches, such as lasso and elastic net regression [52], were also verified. Outliers were screened using Cook’s Distance [48,49], with values exceeding 3.5 times the mean removed, resulting in the exclusion of 6% of the sample (140 out of 2291 participants). Note that this percentage is coherent with the regression modeling literature where habitually 2 to 8% of a sample may be acceptably identified, depending also on the characteristics of the data set and the outlier detection statistic used [53,54,55]. Standard linear modeling assumptions were assessed including residual normality (QQ-plot inspection), homoscedasticity (Breusch–Pagan test), and independence (Durbin–Watson test), and absence of collinearity (Variance Inflation Factor, VIF values < 5).

To gain a deeper understanding of variable associations and to identify distinct profiles within the sample, a clustering analysis was performed. Unlike the regression modeling approach which assumes a single model for all, clusters can reveal subgroups with distinctive characteristics that may either complement or extend the insights obtained from the regression analysis. Prior to clustering, the data suitability was confirmed using the Kaiser–Meyer–Olkin (KMO > 0.60) and Bartlett’s Sphericity (*p* < 0.05) test thresholds. The clustering variables were of the dependent (or pathological) type, including parent burnout, fatigue, anxiety, depression, and insomnia. Then, several algorithms were examined for their capacity to achieve optimal cluster separation and cohesion (k-means, Agglomerative, Birch, DBScan, Spectral), with the spectral clustering algorithm providing the best solution. Cluster means were then calculated for both the defining pathological variables, and all other variables to determine whether clusters based on pathological profiles exhibited distinct, observable differences. Significant differences between clusters were assessed using two sample Student *t*-tests with Bonferroni-corrected *p*-values.

Finally, in order to test the mediation hypotheses (e.g., linking child and parent insomnia to burnout), mediation models were applied within the structural equation modeling framework. Mediation effects were considered significant if the “Indirect Effect” (or mediating path a*b) reached *p* < 0.05. Prior to fitting each model, correlations between the variables involved in the mediation paths were verified.

## 3. Results

### 3.1. Population Sample and General Trends

The final sample consisted of 2291 participants for whom all of the following analyses were conducted. As previously specified, this sample combined the random sample from the general population (*n* = 1409), and a more clinically oriented group (*n* = 882) recruited at a university-affiliated sleep research institute, who sought consultations regarding their child’s sleep disturbances. The overall mean burnout (PBI) score out of a possible total of 132, was 35.2 (SD = 22.5). No significant difference in mean PBI was found between the general sample (M = 34.9, SD = 23.1)) and the clinical group (M = 35.8, SD = 21.4), (Welch *t*(2012.40) = −1.71, *p* = 0.09). Similarly, 21.9% of the overall sample satisfied the PBI criterion for moderate burnout and 6.4% for severe burnout; no significant difference in χ^2^ independence tests (*p* = 0.51 and 0.59) were found between the general and clinical groups as their respective proportions were both within 1% of the overall sample values. In contrast, the clinical group presented higher child sleep disturbance scores (SDSC) with a mean of 49.5 (SD = 10.5) compared to 39.8 (SD = 11.7) in the general sample, (*t*(2187.26) = 22.97, *p* < 0.001). The clinical group also had a higher proportion of mothers with a diagnosed sleep disorder (4.9% versus 0.8%, χ^2^(1) = 35.8, *p* < 0.001), and their children were, on average, 8.8 months younger (*t*(1804.23) = −14.36, *p* < 0.001). The mean age of mothers was 35.4 years (SD = 4.8) and that of fathers was 37.1 years (SD = 5.7); mothers in the clinical group were on average 1.4 years older whereas fathers were 0.6 years younger (both *p*-values < 0.05).

With regard to the assessment of the primary hypotheses of this study using independent univariate tests, first, both a median split two-sample Welch *t*-test (*t* = 11.13, *p* < 0.001, *g* = 0.45) and a Pearson correlation (*r* = 0.29, *p* < 0.001) confirmed that higher levels of child sleep disturbance (SDSC) were associated with higher levels of parental burnout. Second, higher shame scores (PFQ–2) were associated with higher parental burnout scores (*t* = 15.39, *p* < 0.001, *g* = 0.64, and *r* = 0.38, *p* < 0.001). Third, interpreting crying as a call for attachment was associated with higher levels of burnout (*t* = 6.10, *p* < 0.001, *g* = 0.25, and *r* = 0.19, *p* < 0.001); interpreting crying as an expression of pain was significant only in the correlation analysis, and weakly so (*t* = 1.89, *p* = 0.06, *g* = 0.08, and *r* = 0.06, *p* = 0.005). Finally, having more caregivers was not significant in these simple analyses (*p* < 0.05 for both) whereas higher levels of couple conflict were associated with higher levels of burnout (*t* = 7.86, *p* < 0.001, *g* = 0.33, and *r* = 0.10, *p* < 0.001).

### 3.2. Linear Regression Analysis: Variables Associated with and Predictive of Parental Burnout

In this section, a multivariate, model-based analysis (regression) was conducted with the primary objective of quantifying the strength of the association between each variable and parental burnout, while accounting for the effects of the other variables, thereby providing a more ecologically valid and robust framework.

Secondly, this analysis aimed to deepen the understanding of parental burnout through its data-driven component capable of identifying additional variables that may be strongly associated with parental burnout. This would otherwise not have been detected via pre-defined univariate tests used to examine the initial subset of hypotheses (namely, the confirmatory approach).

A significant model equation was obtained (*F*(64, 2086) = 24.04, *p* < 0.001) and all model assumptions (e.g., normality, homoscedasticity, error independence, absence of multicollinearity) were adequately met. Note that all continuous predictor variables were normalized to satisfy said assumptions. All VIF values were below 5. The light outlier filtering approach described in the Methods resulted in the exclusion of 140 participants (6%), hence the modeling results are based on 2151 participants. It was verified that no significant difference was observed between this omitted outlier sample and the remaining sample on primary outcome or demographic variables (e.g., mother burnout, depression, anxiety, insomnia, age, and child age: namely, all two-sample Student *p*-values were found >0.25). The observed *R*^2^ and adjusted *R*^2^ of the most optimal model were 0.43 and 0.41 respectively.

Table 1 provides the results of the predictor set that optimized model fit (or the prediction) of parental (maternal) burnout scores, as measured by the PBI. These are ordered by descending magnitude, with those above the dotted line positively associated with burnout and those below negatively associated. As shown in Table 1, the results replicated the original hypothesis tests. For example, shame and guilt were strongly associated with higher levels of parental burnout, as were child sleep disturbance, interpreting nocturnal cries as an expression of pain (and relatedly, longer durations of nighttime crying), and couple conflict. Additionally, although having more caregivers (more resources) was not significant in the univariate tests, this variable was associated with lower burnout when controlling for other variables. The same pattern was observed for father involvement in nighttime care (more resources available to meet sleep-related caregiving demands).

In addition to quantifying the relative strength of association of each variable, while controlling for the others, this analysis newly identified additional variables that were significantly associated with burnout. For example, Table 1 highlights both intrinsic and extrinsic stressors as risk factors for higher burnout scores, including the belief of being a bad parent, having a child with more siblings, and daytime behavioral problems. The model also highlights other variables associated with parental vulnerability including a difficult childhood, diagnosed sleep disorder/use of hypnotics, older maternal age, disagreements in parenting values, and difficulty in childrearing. Finally, parents who view screen time as a preferable way to calm a child may be exhibiting burnout symptoms (e.g., exhaustion).

Couple satisfaction showed the strongest association with lower burnout outcomes. Other factors of lower but comparable magnitude included practicing a regular pre-sleep or bedtime routine, having more caregivers (e.g., grandparents, aunts/uncles), being an evening chronotype, the mother and father living together, and relatedly, frequent father involvement in nighttime care. Finally, having a child on medication or supplements, allowing the child to use a screen alone, and the parents’ history of prior trauma were also associated with lower burnout scores.

These latter three variables are discussed further in Section 4. The first two may reflect parental relief, whereas the third may indicate the development of coping resources (e.g., post-traumatic growth) and enhanced perspective-taking.

Finally, note that parent psychopathological variables were not included in the regression model as the aim was to identify contextual, individual, and organizational variables associated with parental burnout risk, rather than to explain variance in burnout through common comorbidities. However, fatigue (Pichot), anxiety (STAI), depression (BDI), and parent insomnia (ISI) symptoms were observed to be significantly associated with parental burnout when explored in the model. This finding is further corroborated by the clustering analysis in the following section.

### 3.3. Cluster Analysis: Profiles of Parental Pathological Symptom Combinations and Associated Variables

To gain a deeper understanding of the trends linked to burnout, and to allow for the possibility of sub-models (rather than assuming a single regression model fits all participants equally well), a cluster analysis was performed. First the data were verified as suitable for cluster analysis meeting the KMO test (KMO = 0.80) and Bartlett’s Sphericity test (*p* < 0.001) thresholds (>0.60, <0.05 respectively). Then, an optimal solution balancing parsimony and exhaustivity, with acceptable cluster cohesion and separation was achieved with four clusters (see Supplementary Data S2).

**Figure 1:**
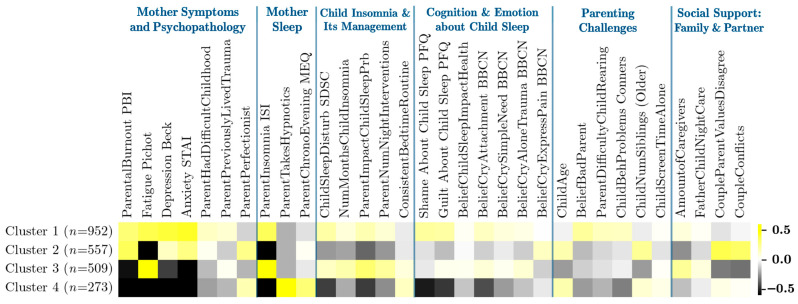
Standardized variable means for each cluster from the cluster analysis, *n* = 2291. *Note*: Variables were first *z*-score standardized with the full *n* = 2291 sample and then averaged. Therefore, colors near white represent a score close to the global average, whereas bright colors represent a cluster above the global average for this variable, and dark colors a cluster below the global average. PBI: Parental Burnout Inventory; STAI: State–Trait Anxiety Inventory; ISI: Insomnia Severity Index; MEQ: Morningness–Eveningness Questionnaire; SDSC: Sleep Disturbance Scale; BBCN: Baby Crying at Night Scale; PFQ: Personal Feelings Questionnaire; Conners: Conners’ 10-item Child Behavior Rating Scale.

Figure 1 displays the mean value for each variable across the four resulting clusters. Note that variables were first *z*-score standardized (with the full sample, *n* = 2291), prior to calculating these means. Therefore, colors near white represent a cluster score (mean) close to the overall *n* = 2291 sample mean ( $\bar{z}$_global_ = 0), whereas bright colors represent a cluster being above the global mean for this variable ( $\bar{z}$_cluster_ > 0), and dark colors indicate a cluster below the global mean ( $\bar{z}$_cluster_ < 0). For ease of interpretation, conceptual grouping labels are provided in blue text on top of Figure 1.

This analysis identified at least two main distinct profiles of Parental Burnout (Cluster 1, *n* = 952 and Cluster 2, *n* = 557) which differed substantially in their associated variables or antecedents. Namely, Cluster 1, the largest group, is characterized by burnout closely related to child sleep disturbance (SDSC), child/parent insomnia, and its management (e.g., frequent night interventions, difficulty in achieving a consistent bedtime routine). They exhibit maladaptive cognitions or emotions related to their child’s sleep, including: high levels of shame and guilt, preoccupation with the impact of sleep deficit impacting on their health, and stressful interpretation of nighttime crying as unfulfilled attachment needs or traumatizing for the child. These parents also experience daytime parenting challenges including more difficult child behavior (Conners), difficulties in childrearing, and discouraging cognitions such as an overall self-perception as a bad parent, compounded by their own insomnia. In addition to their symptoms, these parents show higher average vulnerability traits including a history of difficult childhood experiences and prior trauma (for all previously noted variables, Bonferroni-corrected *p* < 0.05 compared to Cluster 2). Note that Cluster 3, despite reporting above-average prior trauma, exhibited among the lowest burnout levels, which helps clarify why this variable appeared as protective in the regression analysis. Consequently, this demonstrates the added value of a clustering approach allowing several models (i.e., clusters) to provide a deeper understanding of the variable trends.

In contrast to Cluster 1, the parental burnout profile of Cluster 2 (*n* = 557) was not principally characterized by sleep disturbances in children or mothers, but rather by general parenting burden/pressure, lack of assistance, and greater social stressors. That is, while Cluster 2 showed significantly lower symptom scores on sleep-related variables and themes compared to Cluster 1, parents in Cluster 2 exhibited a significantly higher number of children to take care of simultaneously, fewer persons to help (e.g., family members, social support), receive fewer nighttime interventions from fathers, and higher couple tension by both conflict and disagreement in parenting values. Additionally, this cluster also showed significantly higher scores of parent (maternal) perfectionism, an intrinsic source of demands or pressure in a seemingly stress-inducing parental context. Finally, children in this cluster were significantly older on average, which may contribute to differences in daytime parenting challenges, as well as greater child autonomy in sleep (as shown by the lower child sleep disturbance scores observed here, as well as in Cluster 4 which includes older children on average).

Next, Cluster 3 (*n* = 509) comprised a sample that, despite high levels of child and parent insomnia and parent fatigue relative to the global sample average, exhibited significantly lower levels of burnout symptoms (*p* < 0.05 compared to Clusters 1 and 2). This cluster appeared to mitigate the impact of several risk factors observed in Clusters 1 and 2. Namely, parents in Cluster 3 showed adaptive parent beliefs (e.g., low levels of shame, guilt, rarely associating nocturnal cries with infant pain), strong social support (e.g., multiple caregivers, fathers involved at night, low couple conflict), consistency in child rearing/fewer child behavioral problems, regularity in the child’s pre-sleep (or bedtime) routine, and fewer children. Finally, although this cluster exhibited significantly higher levels of parental history of trauma compared with Cluster 2 (*p* < 0.05), the magnitude of this variable was similar to that observed in Cluster 1 (*n.s.*).

Finally, Cluster 4 (*n* = 273) exhibits the lowest levels of parental burnout and overall psychopathological symptoms. Parents in this cluster were the most evening-oriented (HO Chronotype), their children displayed the fewest sleep disturbances and behavioral problems, and there was consistency in the bedtime routines. These parents reported the lowest levels of maladaptive beliefs (shame, guilt, negative interpretations of crying) and had fewer adverse past experiences (their own childhood, fewer traumas). Children in this cluster were older children than those in clusters characterized by parent insomnia (e.g., 1 and 3, *ps* < 0.05). Additionally, this cluster included significantly more individuals, on average, taking hypnotics (e.g., benzodiazepines), compared with the other three clusters (all *ps* < 0.05), which may have contributed to the reduction in measured psychopathological symptoms (e.g., insomnia, anxiety) despite the higher levels of parental perfectionism and couple tension relative to Clusters 1 and 3.

### 3.4. Mediation Analysis: Role of Child Insomnia and Parental Beliefs in Parental Insomnia and Burnout

**Figure 2:**
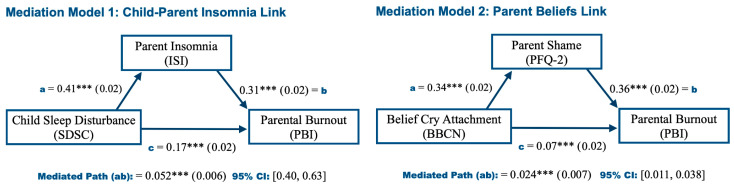
Mediation analyses for the hypotheses of a child–parent insomnia link, and parent beliefs link, *n* = 2291. *Note*. Mediation models were estimated using a structural equation modeling framework to test the hypotheses that child–parent insomnia predicts burnout (Model 1) and that parental beliefs (unmet child needs of attachment, and shame with burnout) predict burnout (Model 2). Significance is indicated by asterisks (***), corresponding to *p* < 0.001), with standard errors shown s in parentheses; 95% CI: 95% Confidence Interval via Bootstrap analysis (1000 draws); *R*^2^ = 0.17 and 0.15 for PBI of Model 1 and 2 respectively. SDSC: Sleep Disturbance Scale; ISI: Insomnia Severity Index; PBI: Parental Burnout Inventory; BBCN: Baby Crying at Night Scale; PFQ–2: Personal Feelings Questionnaire–2.

This final results section presents the mediation analyses conducted to test the hypotheses outlined in the Introduction. Figure 2 displays the path coefficients, their significance (by asterisks) and standard errors (in parentheses) for each of the paths modeled in the two mediation hypotheses tested. Specifically, Model 1 examines child sleep disturbances (insomnia) predicting parental insomnia, which in turn predicts higher levels of burnout, and secondly, Model 2 examines parental beliefs that nocturnal crying is linked to unmet attachment needs predicting higher levels of shame, which subsequently predicts higher levels of burnout. As shown in Figure 2, all paths yielded significant coefficients, including the full mediated path (or Indirect Effect, ab) at *p* < 0.001. These results support the conjectured hypotheses. Finally, in both models, the direct path (between the exogenous and outcome variable) remained significant despite the mediation path (i.e., partial mediation is observed), indicating that the mediator did not fully account for the relationship between said two variables. Thus, these exogenous variables may be associated with additional factors, as suggested by the cluster analysis. For example, the cluster analysis would suggest that Parental Fatigue was associated with SDSC in Model 1 and also linked to Burnout also, whereas Parental Guilt was associated with BBCN in Model 2 and likewise linked to Burnout.

## 4. Discussion

In an effort to understand parental burnout, and following the framework of the Job Demands–Resources model [56], previous research has culminated in a substantial model, the BR^2^ model [1], which conceptualized parental burnout in terms of organizational, relational, and individual factors as risks or resources [12]. However, an important gap in the current theoretical literature of parental burnout concerns the absence of consideration for parents’ “job”, or responsibility in monitoring their child’s sleep wellness/problems and the impact of this responsibility. Moreover, there has been limited research examining this association with an in-depth consideration of the comprehensive biopsychosocial ecosystem surrounding this responsibility remaining limited.

Therefore, the aim of this study was to explore profiles of parental burnout through the lens of child sleep management, in addition to the parameters classically considered in the BR^2^ model. That is, by simultaneously considering psychological, relational, organizational, and sociodemographic factors alongside sleep variables, our approach aimed to provide a more integrated understanding of the mechanisms likely to affect the mental health of mothers of young children. This perspective is particularly important, as early childhood represents a period of heightened vulnerability during which nighttime demands, parental adjustments, and sleep disturbances intersect in pronounced ways.

This study aimed to test several hypotheses situating parental burnout in relation to child sleep management and insomnia, focusing on variables that measure child sleep disturbances, parental psychology (shame, guilt and interpretations of nocturnal crying), and social/relational factors (family, couple support). Given that this research assessed a very wide array of (organizational, individual, relational) variables, it also incorporated data-driven analyses to further advance understanding by identifying additional variables strongly associated with burnout (in comprehensive, multivariate analyses).

First, the primary hypotheses were confirmed, specifically a *Child Sleep Problem/Insomnia Link with Burnout*: (1) mothers of children with poor sleep had significantly higher scores of parental burnout on average; a role of *Parent Emotional Experience regarding their Child’s Sleep Problems*: (2) mothers exhibiting higher levels of shame and guilt regarding their child’s sleep problems exhibited higher average parental burnout scores; as well as a role of *Parent Cognitions in respect to their Child’s Sleep Problems and Nighttime Crying*: (3) related to the previous hypothesis, mothers who interpreted their child’s nocturnal cries as expressions of attachment needs or pain exhibited higher average parental burnout scores; and finally, a *Social/Relationship Resources Link*: (4) mothers with lower social support, from their family, entourage, or partner, had higher average burnout scores. Furthermore, the insomnia and cognitive links were further examined using mediation analyses (see Figure 2) which showed: (a) child insomnia predicted maternal insomnia, which in turn predicted parental burnout; and secondly (b), higher scores on attachment or pain-related crying interpretations predicted higher levels of shame which in turn predicted higher levels of burnout. These findings can be mapped onto the BR^2^ model framework (organizational, relational, individual factors) as follows: hypothesis (1) involves organizational factors, including individual (child) factors, hypothesis (2) and (3) pertain to parent individual factors, and hypothesis (4) pertains to relational factors.

Beyond the scope or usual limitations of confirmatory hypothesis testing, the data-driven analyses allowed for the identification of additional variables that were significantly associated with parental burnout. These are presented in Table 1 for the regression analysis, and in Figure 1 for the clustering analysis. In the regression, for example, organizational variables such as having more siblings were significantly associated with higher burnout, whereas greater regularity in the pre-sleep (bedtime) ritual was associated with lower burnout. Individual-level variables including maladaptive beliefs (of perceiving oneself as a bad parent) and vulnerabilities (older maternal age, history of difficult childhood experiences or a sleep disorder) were associated with higher burnout, while a naturally nocturnal chronotype was associated with lower burnout. Relational variables, including parents living together, greater paternal involvement in nighttime care, higher relationship satisfaction and more caregivers (family/friends) were associated with lower burnout whereas the presence of relationship conflict and value disagreements were associated with higher burnout.

Finally, the clustering analysis demonstrated that a single weighted regression model may not accurately describe every mother–child dyad, and that different profiles of pathology and wellness, associated with different variable patterns, can be observed. Crucially, the cluster analysis demonstrated that at least two models of parental burnout should be distinguished in the literature. We first identified a profile of burnout linked with child insomnia (Cluster 1, *n* = 952) and a profile linked with general parenting stress (Cluster 2, *n* = 557); the former, corroborates the novelty of this research, while the latter aligns with the previous parental burnout research that did not substantially consider the role of child insomnia. The capacity of this analysis to detect cluster-specific trends is evidenced by variables appearing associated with higher levels of symptomatology in one cluster, versus lower in another, or, functioning as additional demands, or conversely, resources, depending on the profile-highlighting distinct profiles. For example, *Previously Lived Trauma* differed between Cluster 1 and Cluster 3. This invites the interpretation of this variable being linked to vulnerability in Cluster 1 versus a source of resilience in Cluster 3. In line with this principle of distinct models, or profiles, the clusters analysis also allowed additional variables to emerge that did not appear significant in the regression analysis because their effects diverged between clusters. For example, Cluster 1 which has younger children on average is associated with higher levels of fatigue, parental burnout, and insomnia (e.g., a potential pathway as identified in Mediation Model 1) versus Cluster 3 included younger children and similar fatigue levels but exhibited the lowest burnout levels; this cluster exhibited higher variables values tied to individual and relational resources).

The cluster analysis made it possible to understand parental burnout through a conceptual grouping of six variables domains: (1) *Maternal Symptoms and Psychopathology*, (2) *Maternal Sleep Dynamics*, (3) *Child Insomnia and Its Management*, (4) *Cognitions and Emotions about Child Sleep*, (5) *Parenting Challenges*, *and* (6) *Social Support (Family, Partner, Entourage).*

### 4.1. Maternal Symptoms and Psychopathology

In relation to the JD-R model [56] of professional burnout, the two distinct profiles of Parental Burnout revealed in this study (Figure 1) can be conceptualized respectively as being predominantly driven by (1) *nighttime parenting demands*, namely child sleep wellness and insomnia management, or (2) *daytime parenting demands.* With the exception of fatigue, as measured by the Pichot scale, and observed in the insomnia-driven burnout profile, in both cases, our analyses revealed that both profiles shared a common comorbidity with depression and anxiety. This association with psycho-affective pathology is consistent with previous studies on burnout in both professional [57] and parental contexts [11]. Fatigue was also identified in Cluster 3, which exhibited a similar level of nighttime parenting demands as Cluster 1 but benefited from appropriate resources, and therefore did not present other symptoms or psychopathologies. Finally, although not strictly considered a psychopathology, the higher levels on average of perfectionism may be conceptualized as additional, self-imposed demands within an already stressful environment, further supporting the burnout profile observed in Cluster 2, which was overwhelmed by daytime parenting demands. Perfectionism has been linked to parental burnout in several studies [58], but may also have adaptive aspects [59]. Conversely, perfectionism was not observed in either group presenting significantly higher levels of fatigue, a finding that warrants further investigation, for example to examine whether its deactivation may function as a successful defense mechanism, or to distinguish between maladaptive and adaptive forms of perfectionism [60,61].

### 4.2. Mother Sleep Dynamics

Our analyses demonstrated that maternal insomnia, in both cases (Clusters 1 and 3), was tied to child sleep disturbances and insomnia, regardless of whether significant levels of parental burnout were observed, present in the former, absent in the latter. This finding underscores the crucial role of sleep well-being in the mother–child dyad. The regression analysis further suggests that parents with a diagnosed sleep disorder are more likely to report higher burnout symptomatology on average. Therefore, the differentiation between Clusters 1 and 3 may be explained by the BR^2^ model [2,5], insofar as sleep deficit may reduce parenting resources, thereby increasing the overall likelihood of parental burnout. Thirdly, our analyses (both the clustering and regression) suggest that parents with an evening chronotype are less likely to exhibit significant levels of parental burnout, which may reflect greater nighttime resources.

### 4.3. Child Insomnia and Its Management

In our analyses, for both Clusters (1 and 3) the management of child insomnia (presence of child sleep disturbances, more frequent nighttime interventions) was associated with significantly higher levels of parental fatigue. In turn, parental fatigue was associated with the burnout profile predominantly linked to child insomnia, but not with the burnout profile primarily linked to daytime parenting burden and stressors. Across both the regression and cluster analyses, this study highlights the important and protective role that a consistent bedtime routine may play in managing child insomnia and therefore parental burnout (supported by the mediation analysis). Specifically, the regression analysis found that on average consistent bedtime routines were associated with lower levels of parental burnout. The cluster analyses shed further light on this pattern: both burnout clusters exhibited lower consistency in bedtime routines, whereas the cluster characterized by low burnout despite high child sleep disturbance demonstrated significantly greater consistency in bedtime routines. In line with developmental and cognitive-behavioral therapy research, a stable bedtime routine may facilitate the acquisition of self-soothing skills and independent sleep [27]. By reducing children’s nighttime demands, and thereby preserving parental sleep as a resource, the prevention of insomnia in young children can be conceptualized within the BR^2^ model as a protective factor against parental burnout.

### 4.4. Cognitions and Emotions About Child Sleep

Children in Clusters 1 and 3 exhibited clinically significant sleep disturbances (pathological SDSC scores); however, only mothers in cluster 1 reported parental burnout. This difference appears to be explained not only by higher levels of anxiety and depression in cluster 1, but more importantly by stronger dysfunctional beliefs [36] and greater feelings of guilt and shame related to their child’s nocturnal crying. In line with the CEMYCI framework [27], these findings suggest that parental burnout is not solely determined by the level of behavioral demands (here, nighttime child solicitations), but rather by parents’ cognitive and emotional appraisals, which may constitute the key discriminating factors in the presence or absence of parental burnout.

### 4.5. Parenting Challenges

This study, as illustrated by the two distinct parental burnout profiles that emerged, emphasizes that different dynamics are associated with nighttime versus daytime parenting demands (or challenges). The previous subsections have insofar focused on the nighttime parenting demands. Overall, the regression analyses indicated that child behavioral problems and difficulty in managing them were indeed significantly associated with higher levels of parental burnout whilst controlling for other variables. In contrast, the clustering analyses (e.g., Cluster 2) revealed that parenting more generally can lead to higher levels of parental burnout even without significantly elevated child behavioral problems. Notably, having more children is a significant risk factor (as also in [2,10]) especially when combined with self-imposed demands (pressure) such as a perfectionistic personality trait. This analysis also suggests, or corroborates the previous research, that older children (in the 0.5–6 year age range) may present different demands that are exhausting but not necessarily “problematic” [62]. In contrast, the burnout profile linked to child insomnia showed the highest reports of difficulty in child rearing and behavioral problems. According to the JD-R framework, this may suggest that mothers experiencing insomnia and fatigue possessing fewer resources to meet daytime parenting demands without restorative sleep. This leads to the consideration of how parents might take breaks (or naps) to replenish their resources [63,64]. Indeed, our analyses (notably regression) identified child digital screen time as significantly associated with lower parental burnout in the regression modeling. However, the clustering analyses indicate that this variable is less decisive than it initially appears, and may be beneficial only when carefully considered. For example, a previous study identified evening child screen time as associated with greater child sleep disturbances [65]; therefore, parental breaks facilitated through child screen use should be preferably used in moderation and earlier in the day.

### 4.6. Social Support: Family, Partner, Entourage

Our analyses highlighted the important role of social support in managing parental burnout. A significant protective effect of having more caregivers (partner, extended family, entourage) and greater father nighttime interventions was observed. These factors can be interpreted as additional external resources, or opportunities to replenish personal resources [63,64] (preferable to the aforementioned use of child digital screen times). Couple satisfaction emerged as the variable most strongly associated with lower levels of parental burnout, functioning either as an inspirational resource or a reflection of a mother’s perceived satisfaction with sharing parenting responsibilities. Conversely, couple conflict and disagreement in parenting values represents additional demands or stressors related to childrearing, which were indeed significantly associated with higher levels of burnout. However, returning to the role of positive social support and the absence of couple conflict, particularly for the insomnia parental burnout profile (Cluster 1), suggests that on average, these factors may not be sufficient to overcome the cognitive and emotional burdens. Therefore, cognitive-behavioral therapy aimed at improving mental health, self and child-related perceptions, and strengthening behavioral management skills, appears to remain crucial for mothers even with seemingly supportive social environments.

### 4.7. Burnout Risk May Be Further Contextualized by Individual-Level Interactions Between the Child and Parent

Individual-level variables related to both the mother and the child are important to take into account, particularly their interactions. For example, the analyses showed that daytime parenting difficulties (e.g., child behavioral problems, perceived childrearing difficulty), as additional stressors, were associated with parental burnout linked to child insomnia (Cluster 1, and the regression). Mothers with a difficult childhood, or unresolved prior trauma may be more likely to experience difficulties in daytime parenting (Cluster 1). Moreover, the regression analysis indicated that older mothers may be more susceptible to exhibiting higher levels of burnout symptoms. Mothers with a diagnosed sleep disorder appear to represent a vulnerable group. The use of hypnotics may function as a double-edged coping strategy: it may help manage their own sleep and anxiety symptoms, and their child’s sleep problems.

### 4.8. Perspectives: Situating Sleep Disturbance Variables with Biological Mechanisms

Integrating all of our findings highlights three complementary explanatory axes, the first of which involves a central physiological mechanism. This axis refers to the role of chronic parental fatigue, resulting from child sleep disturbances, as a driving factor in parental burnout syndrome. Repeated nighttime awakenings, particularly when embedded in an unequal distribution of nighttime caregiving, constitute a persistent source of sleep deprivation likely to durably impair parental adaptive capacities.

At the neurobiological level, the literature suggests a bidirectional relationship between chronic sleep restriction and dysregulation of the hypothalamic–pituitary–adrenal (HPA) axis, involving alterations in cortisol circadian rhythms and stress reactivity [3,22]. This disruption of stress-response systems may contribute to increased vulnerability to burnout by promoting sustained physiological activation and the gradual depletion of energetic and emotional resources. These neuroendocrine mechanisms appear to be closely intertwined with processes of emotional dysregulation, which may constitute a key mediator between parental sleep debt and burnout [66]. Under conditions of chronic sleep deprivation, the capacity to regulate negative affect, tolerate frustration, and maintain cognitive flexibility tends to diminish, progressively compromising emotional adjustment to the daily demands of parenting [67]. From this perspective, fatigue induced by child sleep disturbances is not limited to transient discomfort, but would instead contribute to a gradual erosion of parental resources, reducing the psychological availability necessary to respond appropriately and sensitively to the child’s needs [21]. Thus, our findings support the hypothesis that the impact of child sleep disturbances on parental sleep constitutes a major etiological factor in parental burnout, particularly when this disruption is prolonged and accompanied by an unequal distribution of nighttime caregiving between parents.

A second psychological axis integrates maternal anxiety, depression, and fatigue, which shape how nighttime disruptions are experienced and managed. Finally, a relational and systemic axis encompasses marital dynamics, family composition, and the quality of coparenting, all of which influence how nighttime stress is distributed and buffered. Within this latter axis, the sharing of nighttime caregiving between parents emerges as an important protective resource that may reduce the risk of maternal burnout. This integrative perspective, consistent with recent models of parental burnout [1], underscores that no single factor is sufficient to trigger exhaustion; rather, it is the cumulative interactions among these factors that warrant clinical attention.

It is important to note that parental exhaustion is not solely attributable to the nighttime burden of managing children. In fact, in Cluster 3, where parental burnout was absent, children and parents experienced poor sleep, however mothers exhibited little or no dysfunctional emotions and cognitions related to their child’s sleep (unlike mothers in Cluster 1). In this Cluster, children were less disruptive during the day and parents received greater social support. Another distinction between Clusters 1 and 3 concerns the mothers’ history of trauma or difficult childhoods experiences. These results suggest that parental exhaustion does not arise exclusively from sleep-related demands/resources, but also from cognitive and emotional factors, likely linked to a traumatic history.

### 4.9. Clinical Implications

The first year of a child’s life constitutes a critical period, during which nighttime awakenings are frequent and parental resources are limited. The results of this study stress the importance of supporting mothers in developing adaptive self-beliefs, self-perceptions, and appropriate interpretations of infant nocturnal crying [68]. Strategies aimed at improving the daytime management of child behavioral difficulties may also alleviate stress and fatigue, thereby facilitating nighttime caregiving, especially for mothers with a morning chronotype [69]. Thirdly, encouraging mothers to seek support either from family members, friends, or the co-parent, is significantly associated with lower parental burnout [1]. Furthermore, early paternal involvement in nighttime caregiving appears to be an important protective factor, consistent with previous research demonstrating its beneficial effects on maternal sleep and couple functioning [70,71]. Conversely, the absence of paternal support may reinforce caregiving asymmetries and consolidate patterns in which mothers assume sole responsibility for nighttime care, making any subsequent redistribution more challenging [72,73]. Therapists may also consider assessing and supporting the overall quality of the couple’s relationship, which likewise emerged as a variable strongly associated with lower burnout levels in the regression modeling. Finally, although moderate child screen time alone was on average significantly associated with lower levels of burnout, this should not be considered as an optimal strategy. Rather, it may highlight the broader importance of ensuring that mothers have opportunities for energy recovery [63,64] in order to mitigate exhaustion.

### 4.10. Limitations and Future Research Directions

This study highlights the complex interactions between children’s sleep disturbances, the nocturnal caregiving burden assumed by parents, and vulnerabilities specific to mothers. However, several methodological considerations call for a cautious interpretation of these findings.

Some limitations are inherent to the chosen research design. First, the cross-sectional nature of the data precludes firm conclusions regarding the directionality of the observed relationships. It is plausible that children’s sleep disturbances and maternal nocturnal caregiving burden contribute to parental exhaustion; however, the reverse hypothesis is equally credible. Mothers who are already exhausted may experience greater difficulty soothing their child, maintaining stable routines, or implementing effective regulatory strategies. This ambiguity does not invalidate the observed associations but limits their causal interpretability. Future work further examining and specifying the directionality of the identified relationships would be important for advancing the domain, and for example, of the mediation models examined herein. These works could also consider adding covariates within the mediation modeling framework.

Furthermore, all information regarding the child’s sleep and the sharing of nocturnal caregiving responsibilities relied on maternal self-report. Mothers’ emotional states may have influenced their perception of nighttime awakenings, potentially leading to an overestimation or underestimation of difficulties. The absence of more objective measures, such as actigraphy, or complementary reports (e.g., fathers or clinicians) therefore limits the precision of the data collected. Furthermore, this study included only mothers, which represents a notable selection bias as parental burnout can also affect fathers. Lastly, a few scales (namely, the MEQ) resulted in weaker Cronbach’s α values (0.60), which merits further work to confirm their association with these outcomes found herein.

The fact that the (clinical) subsample consisted exclusively of mothers seeking consultation for their child’s sleep disorder enhances the ecological validity of the findings but restricts their generalizability. The processes identified may differ in the general population or among fathers.

Finally, several key dimensions of the BR^2^ model were not assessed, such as parental self-efficacy and emotional regulation skills. The absence of these variables prevents a comprehensive representation of the balance between risks and resources and excludes potentially protective or aggravating mechanisms. In addition, several variables were not measured with standardized scales, which may have reduced their analytical sensitivity and warrant caution in interpreting their contribution. These limitations provide important avenues for future research, particularly within longitudinal study designs.

## 5. Conclusions

This study, with a robust sample size and data driven analyses, resulted in two distinct profiles of parental burnout which can be hypothesized as two main subtypes: the first corresponds to the form traditionally described in the literature; the second, identified here, arises from high nighttime demands from children, which deplete parental sleep resources. Given that sleep disorders affect a substantial proportion of children, it is crucial to consider not only daytime factors but also nighttime factors in the assessment and management of parental burnout. Future research with longitudinal designs would be instrumental in further evaluating and nuancing these novel findings.

## Figures and Tables

**Table 1:** Linear regression model for predicting the degree of Parental Burnout (PBI), *n* = 2151.

	*β*	CI	*t*	*p*
Shame About Child Sleep (PFQ)	0.21	[0.17, 0.26]	9.25	<0.001
Belief Bad Parent	0.17	[0.14, 0.21]	9.60	<0.001
Child Number of Siblings (Older)	0.12	[0.08, 0.16]	5.78	<0.001
Child Behavioral Problems (Conners)	0.11	[0.07, 0.15]	5.15	<0.001
Child Sleep Disturbance (SDSC)	0.09	[0.04, 0.14]	3.61	<0.001
Parent Had Difficult Childhood	0.08	[0.04, 0.11]	4.17	<0.001
Couple Parent Values Disagree	0.08	[0.03, 0.13]	3.20	0.001
Adult Difficulty Child Rearing	0.06	[0.02, 0.1]	3.14	0.002
Guilt About Child Sleep (PFQ)	0.06	[0.02, 0.11]	2.75	0.006
Couple Conflicts	0.06	[0.01, 0.11]	2.40	0.02
Parent Takes Hypnotics	0.05	[0.02, 0.08]	2.98	0.003
Mother Age	0.04	[0.01, 0.08]	2.48	0.01
Parent Diagnosed Sleep Disorder	0.04	[0.01, 0.07]	2.24	0.02
Parent Interprets Screen Calming	0.04	[0.01, 0.08]	2.22	0.03
Belief Cry Express Pain (BBCN)	0.04	[0.003, 0.07]	2.16	0.03
Child Cries for Long Durations	0.04	[0.001, 0.07]	2.02	0.04
Couple Satisfaction	−0.20	[−0.24, −0.15]	−8.88	<0.001
Parent Chronotype Evening (MEQ)	−0.06	[−0.09, −0.02]	−3.50	<0.001
Consistency Bedtime Routine	−0.06	[−0.09, −0.02]	−3.46	0.001
Child Screen Time Alone	−0.05	[−0.09, −0.02]	−2.98	0.003
Mother Father Living Together	−0.05	[−0.09, −0.02]	−2.85	0.004
Amount of Caregivers	−0.05	[−0.09, −0.02]	−2.80	0.005
Father Child Night Care	−0.05	[−0.09, −0.01]	−2.19	0.03
Child Medication or Supplement	−0.05	[−0.10, −0.01]	−2.05	0.04
Adult Previously Lived Trauma	−0.04	[−0.07, −0.01]	−2.23	0.03

*Note*: *β*: standardized beta coefficient; CI: 95% confidence interval for *β*. Variables above the dotted line are associated with higher parental (mother) burnout, whereas those below are associated with lower burnout. Unless otherwise specified, all “parent” variables pertain to the mother. Non-significant variables (*p* > 0.05) are not included in order to ease readability.

## Data Availability

The dataset which contains sensitive patient information is not posted in a public repository due to legal and ethical constraints. However, it may be requested for research purposes via a data sharing agreement by contacting the corresponding author.

## Supplementary Materials

The following supporting information can be downloaded at: https://www.mdpi.com/article/10.3390/children13030394/s1, Supplementary Data S1 (a): Table providing additional information on the variables measured in this study and their scoring. Supplementary Data S2 (b): First and second principal component values for each participant (x,y coordinates) and their cluster (color) for the four-cluster solution obtained in Figure 1 of the article.

## Abbreviations

The following abbreviations are used in this manuscript:

BBCN	Baby Crying at Night Scale
BDI	Beck Depression Inventory
ISI	Insomnia Severity Index
KMO	Kaiser-Meyer-Olkin
MEQ	Morningness–Eveningness Questionnaire
PBI	Parental Burnout Inventory
PFQ	Personal Feelings Questionnaire
SDSC	Sleep Disturbance Scale for Children
STAI	State–Trait Anxiety Inventory
VIF	Variance Inflation Factor
