# Supplementary material for: Revising Parental Burnout Theory: Toward a Differentiation of Sleep-Related Burnout Subtypes
Source: Children (Basel). 2026 Mar 12;13(3):394. doi: 10.3390/children13030394 (PMC13024748; doi:10.3390/children13030394)

Article

Revising Parental Burnout Theory: Toward a Differentiation of Sleep-Related Burnout Subtypes

Royce Anders, Agnès Breton, Florian Lecuelle, Mélanie Havy, Lisa Brunel, Marie-Paule Gustin, Patricia Franco and Benjamin Putois

Supplementary Materials

Supplementary Data S1

Additional details on measured variables

Variable Label	Description
Parental Burnout	Parental Burnout Inventory (PBI) score – Mother
Fatigue	Parent Fatigue score from Pichot's Fatigue scale – Mother
Depression	Parent Beck Depression Inventory BDI-13 score – Mother
Anxiety	Parent State-Trait-Anxiety Inventory STAI Y-A et Y-B score – Mother
Parent Insomnia	Insomnia Severity Index (ISI) – Mother
Parent Shame	Personal Feelings Questionnaire (PFQ-2) Shame subscale score – Mother
Parent Guilt	Personal Feelings Questionnaire (PFQ-2) Guilt subscale score – Mother
Parent Chrono Evening	Parent Morning-Eveningness Questionnaire (MEQ) score – Mother
Parent Takes Hypnotics	Binary (self-reported) – Mother
Parent Had Difficult Childhood	Mother extent of difficulty in % (self-reported)
Parent Previously Lived Trauma	Mother impact of lived/witnessed trauma in % (self-reported)
Parent Impact Child Sleep Prb	Degree to which considers impact by child's sleep prb % (self-rep)
Mother Age	In years
Mother Education Level	< high-school / high-school / undergraduate / master / doctorate
Mother Employment Status	Unemployed or other / sick leave / night work / part-time / full-time
Father Age	In years
Father Education Level	< high-school / high-school / undergraduate / master / doctorate
Father Employment Status	Unemployed or other / sick leave / night work / part-time / full-time
Mother Father Living Together	Binary
Relationship Status with Father	Binary Divorced or considers self separated versus together
Child Age	In months (calculated from date of birth)
Child Sex	Binary (female=0, male=1)
Child Height Weight	Ratio Height (cm) to weight (kg)
Child Num Siblings (Older)	Number of siblings older than the child that is 0 to 5 years old
Child Sleep Disturbance	Sleep Disturbance Scale for Children (SDSC) total score
Child Behavior Problems	Conners' 10-item also referred to as the Conners' Parent Rating Scale (CPRS)
Child Screen Time Alone	Binary Child is more often with an adult or alone during (TV, tablet, etc.)
Child Number of Night Awakenings	1 / 2 / 3 / 4 / >4
Child Bedtime	Average hour at which child is put to bed

Child Biological disorder	Binary
Cry For Attachment	Interpretation of nocturnal cries as unmet attachment needs (BBCN)
Cry Simple Need	Interpretation of nocturnal cries as simple intrinsic child need (BBCN)
Cry Express Pain	Interpretation of nocturnal cries as child is in pain (BBCN)
Cry Alone is Traumatizing	Interpretation of nocturnal cries as traumatizing to cry alone (BBCN)
Night Crying Long Time	Sum of 3 binary variables (Child cries >3 hrs, inconsolable, often)
Night Crying Waking Others	Sum of 3 binary variables (Waking parents, other family members,neighbors)
Num Months Child Insomnia	Number of months parent considers child has had an insomnia problem
Adult Num Night Interventions	Number of night interventions of 15 types total (binary) from the BISQ
Consistency Bedtime Routine	Number of days per week on average the child has the same pre-sleep routine
Father Child Night Care	Likert 5-item (low scores mother mostly, 0 equal, high scores father mostly)
Father Child Day Care	Likert 5-item (low scores mother mostly, 0 equal, high scores father mostly)
Adult Difficulty Child Rearing	Sum of 4 binary variables (obedience, saying no,imposing rules,limit-setting)
Mother Domestic Tasks	Degree of participation in % (self-reported)
Father Domestic Tasks	Degree of participation in % (self-reported)
Couple Satisfaction	Degree of satisfaction in % (self-reported)
Couple Parent Values Disagree	Degree of disagreement in % (self-reported)
Couple Conflict	Degree of conflict in % (self-reported)
Belief Bad Parent	Degree to which considers self a bad parent % (self-reported)
Belief Child Sleep Prb Impact Health	Degree to which considers child sleep prob impacts child health % (s-r)
Parent Perfectionist	Degree to which considers self a perfectionist % (self-reported)
Mother's Capacity to Delegate tasks	Degree to which considers self effective in delegating % (self-reported)

Supplementary Data S2

First and second principal component values for each participant (x, y coordinates) and their cluster (color) for the four-cluster solution obtained in Figure 1 of the article (n = 2291).

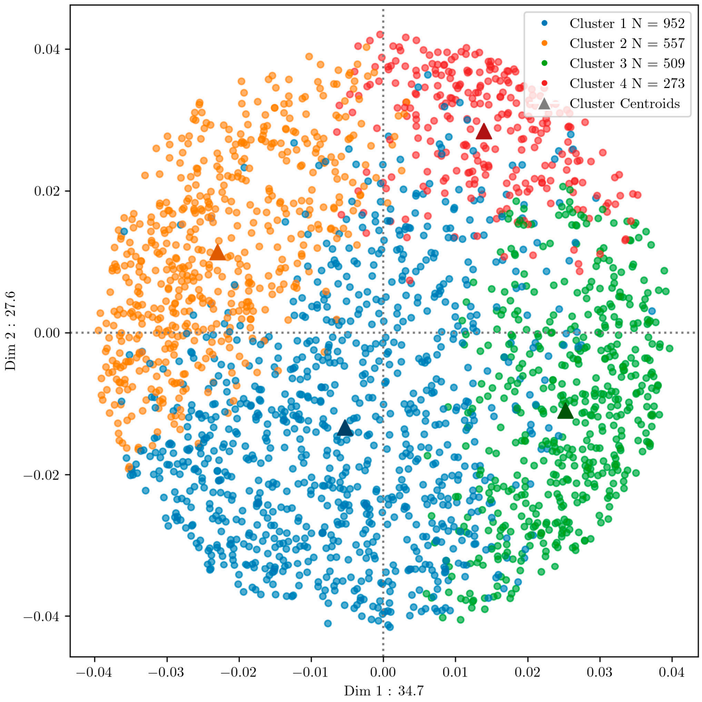

Supplement: Supplementary file 1 [file children-13-00394-s001.zip › children-4183125-supplementary.pdf]
